# A Rare Case of Apixaban-Related Spontaneous Perinephric Hematoma Complicated by Multiorgan Dysfunction

**DOI:** 10.7759/cureus.87612

**Published:** 2025-07-09

**Authors:** Dhayananth Rattaipalivalasu Saravanan, Manogna Pendyala, Mansi Jain, Purnoor Kaur, Salil Avasthi

**Affiliations:** 1 Internal Medicine, Mercy Health St. Vincent Medical Center, Toledo, USA; 2 Pulmonary and Critical Care Medicine, Mercy Health St. Vincent Medical Center, Toledo, USA

**Keywords:** abdominal pain diagnosis, acute kidney injury, anticoagulant adverse effects, apixaban complication, imaging in nephrology, non-traumatic hematoma, perinephric hemorrhage, renal hematoma, retroperitoneal bleeding, spontaneous bleeding

## Abstract

Spontaneous perinephric hematomas are rare but potentially life-threatening events, often associated with anticoagulation therapy. We report a case of a 63-year-old male patient on apixaban for paroxysmal atrial fibrillation who presented with acute abdominal and flank pain and was found to have a large left-sided perinephric hematoma. His clinical course was complicated by acute kidney injury, ileus, respiratory failure requiring mechanical ventilation, and eventual death from hypoxic respiratory failure and cardiac arrest. This case underscores the diagnostic and therapeutic challenges in managing spontaneous retroperitoneal hemorrhage, especially in the setting of anticoagulation, and highlights the importance of early recognition and multidisciplinary management.

## Introduction

Spontaneous perinephric hematoma (SPH), also known as Wunderlich syndrome, refers to a rare but potentially life-threatening condition characterized by spontaneous bleeding into the subcapsular or perirenal space in the absence of trauma. First described by Wunderlich in 1856, it is classically associated with Lenk’s triad: acute flank pain, palpable mass, and hypovolemic shock. However, the presentation is often nonspecific, with flank or abdominal pain being the most consistent symptom [1]. The etiologies of SPH are diverse. In a meta-analysis by Zhang et al., the most common causes were renal neoplasms (61.5%), vascular disorders (17%), and infections (2.4%), with approximately 6.7% of cases considered idiopathic [2]. More recently, the use of anticoagulation, particularly direct oral anticoagulants (DOACs), has been increasingly implicated as a cause of non-traumatic perinephric hemorrhage [3,4]. DOACs, including the factor Xa inhibitors apixaban, rivaroxaban, and edoxaban, are commonly used for stroke prevention in atrial fibrillation and for treatment or prevention of venous thromboembolism. While they offer favorable safety profiles compared to vitamin K antagonists, their use is not without risk. Several case reports have documented spontaneous renal and retroperitoneal hemorrhages associated with these agents, often in the absence of any trauma or structural renal abnormalities [5-8]. Management of SPH varies based on hemodynamic status and the underlying cause. Conservative management is preferred in hemodynamically stable patients without ongoing bleeding, while embolization or surgery may be indicated in unstable patients or those with active arterial bleeding [9,10]. The presence of comorbidities such as renal insufficiency, heart failure, or intra-abdominal complications can significantly complicate the clinical course and outcomes. This report details a fatal case of SPH in a patient on apixaban for atrial fibrillation, complicated by acute kidney injury (AKI), ileus, respiratory failure, and eventual death. It aims to highlight the diagnostic and management challenges posed by this rare but severe entity. 

## Case presentation

A 63-year-old man with a complex cardiovascular history presented to the emergency department with acute abdominal and left flank pain. The pain was sudden in onset, woke him from sleep early in the morning, and was described as moderate to severe in intensity, dull and aching in nature, radiating posteriorly toward the back. It was predominantly localized to the left lower quadrant and flank. The patient also reported associated nausea and gross hematuria, but there was no vomiting, fever, dysuria, increased urinary frequency, chest pain, shortness of breath, lightheadedness, visual changes, or changes in bowel habits. His past medical history included paroxysmal atrial fibrillation, recently started on apixaban (Eliquis) 5mg twice daily one month prior, rate-controlled with extended-release diltiazem (Cardizem CD) 120 mg once daily, a prior cerebrovascular accident on clopidogrel (Plavix) 75 mg once daily, essential hypertension managed with valsartan (Diovan) 80 mg once daily, dyslipidemia, and a history of mitral annuloplasty with closure of an atrial septal defect. Social history was notable for moderate alcohol intake, approximately 14 standard drinks per week (equivalent to approximately 196 grams of ethanol per week), with no history of tobacco or illicit drug use.

On initial evaluation, the patient was alert but appeared uncomfortable. He was tachycardic with a heart rate of 110 beats per minute, blood pressure was 124/80 mmHg, and he was afebrile with a temperature of 98°F (36.7°C). Laboratory workup (Tables 1, 2) revealed AKI (serum creatinine 2.7 mg/dL, baseline 0.9-1.2 mg/dL), lactic acidosis (lactate 6.2 mmol/L), leukocytosis (WBC 15.4 x10⁹/L), an international normalized ratio (INR) of 1.3, and normocytic anemia (hemoglobin 10.4 g/dL with baseline 12 g/dl around four weeks ago). Urinalysis was notable for significant hematuria (Table 3). 

**Table 1:** Complete Metabolic Profile upon Admission

Test	Result	Normal Range	Units
Sodium	141	135–145	mmol/L
Potassium	5.2	3.5–5.0	mmol/L
Chloride	101	98–107	mmol/L
Carbon Dioxide	19	22–29	mmol/L
Blood Urea Nitrogen	29	7–20	mg/dL
Creatinine	2.7	0.6–1.3	mg/dL
Anion Gap	21	8–16	mmol/L
Estimated Glomerular Filtration Rate	26	≥60	mL/min/1.73m²
Glucose	215	70–110	mg/dL
Calcium	9.4	8.5–10.5	mg/dL
Albumin/Globulin Ratio	1.5	1.2–2.2	-
Total Protein	6.9	6.0–8.3	g/dL
Troponin (High Sensitivity)	27	<14	ng/L
Albumin	4.1	3.5–5.0	g/dL
Alkaline Phosphatase	74	44–147	U/L
Alanine Aminotransferase (ALT)	19	7–56	U/L
Aspartate Aminotransferase (AST)	23	10–40	U/L
Total Bilirubin	0.8	0.1–1.2	mg/dL
Lipase	35	0–160	U/L

**Table 2:** Complete Blood Count upon Admission

Test	Result	Normal Range	Units
White Blood Cell Count	15.4	4.0–11.0	×10³/μL
Red Blood Cell Count	3.94	4.7–6.1 (M), 4.2–5.4 (F)	×10⁶/μL
Hemoglobin Concentration	10.4	13.5–17.5 (M), 12.0–15.5 (F)	g/dL
Hematocrit	32.6	41–53 (M), 36–46 (F)	%
Mean Corpuscular Volume (MCV)	82.7	80–100	fL
Mean Corpuscular Hemoglobin (MCH)	26.4	27–33	pg
Mean Corpuscular Hemoglobin Conc.	31.9	32–36	g/dL
Mean Platelet Volume (MPV)	9.9	7.5–11.5	fL
Red Cell Distribution Width (RDW)	15.8	11.5–14.5	%
Platelet Count	275	150–450	×10³/μL
Neutrophil Percentage	94	40–60	%
Lymphocyte Percentage	3	20–40	%
Monocyte Percentage	2	2–8	%
Eosinophil Percentage	0	1–4	%
Basophil Percentage	0	0–1	%
Neutrophil Absolute Count	14.48	1.8–7.0	×10³/μL
Lymphocyte Absolute Count	0.46	1.0–3.0	×10³/μL
Monocyte Absolute Count	0.31	0.2–0.8	×10³/μL
Eosinophil Absolute Count	0.00	0.0–0.5	×10³/μL
Basophil Absolute Count	0.00	0.0–0.2	×10³/μL
Immature Granulocytes Percentage	1	0–0.4	%
Immature Granulocytes Absolute Count	0.15	0.0–0.3	×10³/μL
Nucleated Red Blood Cells (Automated)	0.0	0.0	×10³/μL
Morphology	Anisocytosis Present, 1+ Elliptocytes	-	-

**Table 3:** Urinalysis

Parameter	Result	Reference Range
Color	Yellow	-
Turbidity	Clear	-
Glucose (Urine)	Negative	Negative
Bilirubin (Urine)	Negative	Negative
Ketones (Urine)	Trace	Negative
Specific Gravity	1.064	1.005–1.030
Protein (Urine)	2+	Negative to Trace
Urobilinogen	Normal	0.2–1.0 EU/dL
Nitrite (Urine)	Negative	Negative
Leukocyte Esterase	Negative	Negative
pH (Urine)	5.0	4.5–8.0
Casts	5 to 10 hyaline	0–5 / LPF (non-centrifuged)
WBC (Urine)	2 to 5 / HPF	0–5 / HPF
RBC (Urine)	20 to 50 / HPF	0–4 / HPF
Epithelial Cells	0 to 2	0–5 / HPF
Bacteria	None	None
Urine Hemoglobin	Large	Negative

**Figure 1:**
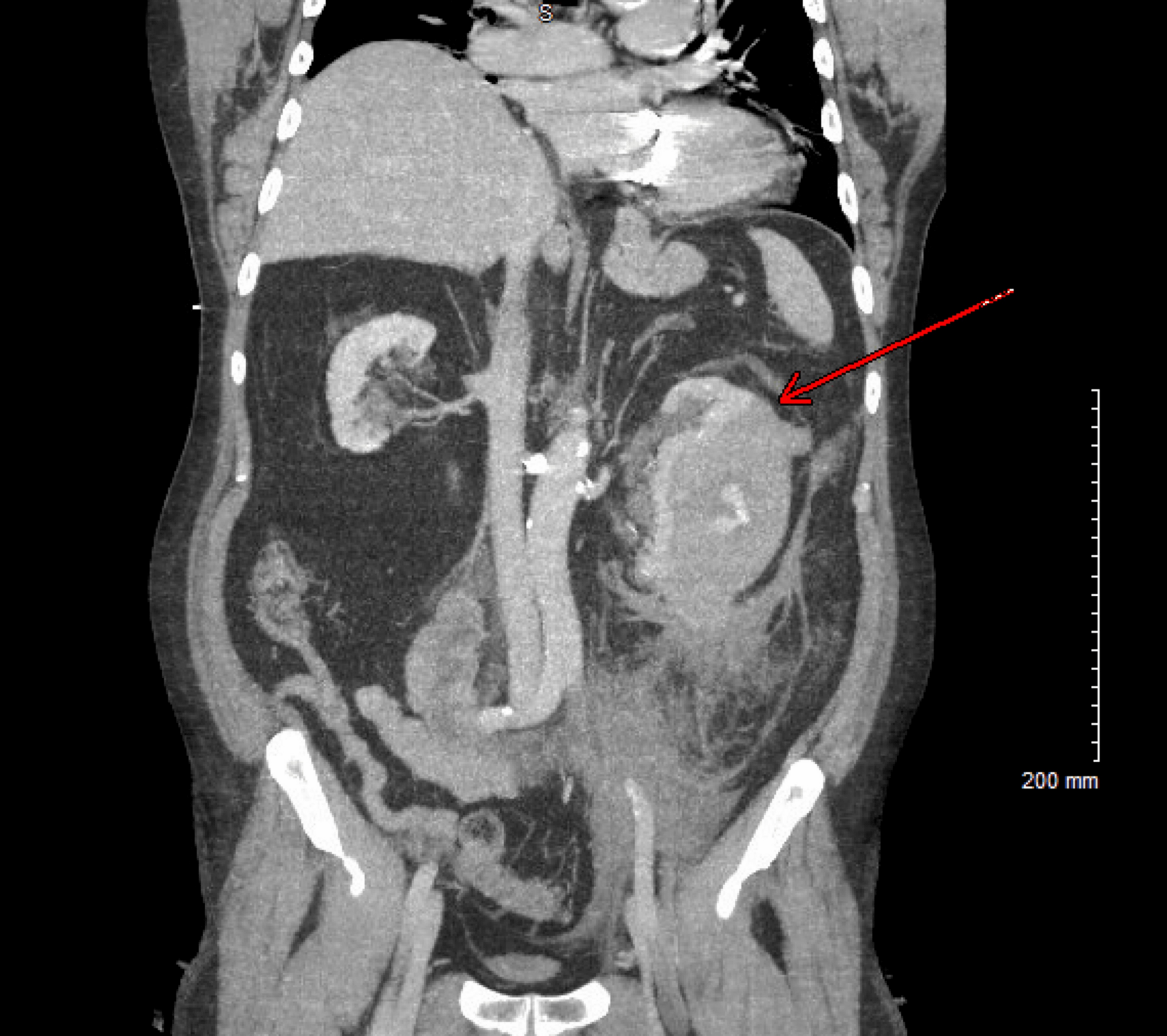
CTA of the Chest, Abdomen, and Pelvis Showing Large Left Perinephric Hematoma With Retroperitoneal Extension (Red Arrow)

The chest radiograph was unremarkable. Given the concerning presentation and abnormal labs, CTA of the chest, abdomen, and pelvis was performed (Figure 1) and revealed a large, acute left perinephric hematoma measuring approximately 138 x 69 mm with hemorrhage extending into the retroperitoneal space.

Apixaban was promptly withheld, and the patient received reversal therapy with activated prothrombin complex concentrate (Balfaxar). He was given two liters of intravenous fluids for resuscitation. Urology was consulted and recommended against surgical intervention due to the risk of nephrectomy associated with hematoma evacuation. Interventional radiology was consulted for possible embolization, but they advised against it due to the diminutive nature of the segmental renal arteries and the limited potential benefit of embolization in alleviating the hematoma’s mass effect. The consensus was to proceed with close monitoring and supportive care in the medical ICU.

Over the following days, the patient’s condition remained tenuous. He developed intermittent episodes of atrial fibrillation with a rapid ventricular response (heart rate up to 160 beats/minute), managed with intravenous metoprolol. His hematuria gradually resolved, and serial hemoglobin measurements showed stability. However, the patient’s renal function worsened despite volume resuscitation, attributed to multifactorial causes, including renal hypoperfusion, possible obstructive effects of the hematoma, contrast exposure, and use of an angiotensin receptor blocker. Nephrology was consulted and recommended conservative management initially. 

By day 3 of hospitalization, the patient developed acute hypercapnic respiratory failure, likely due to reduced ventilatory capacity from abdominal distension and pain-induced hypoventilation. The patient was started on intermittent non-invasive ventilation using BiPAP. Ileus developed soon after, possibly secondary to intra-abdominal hematoma compression and opioid analgesic use. 

**Figure 2:**
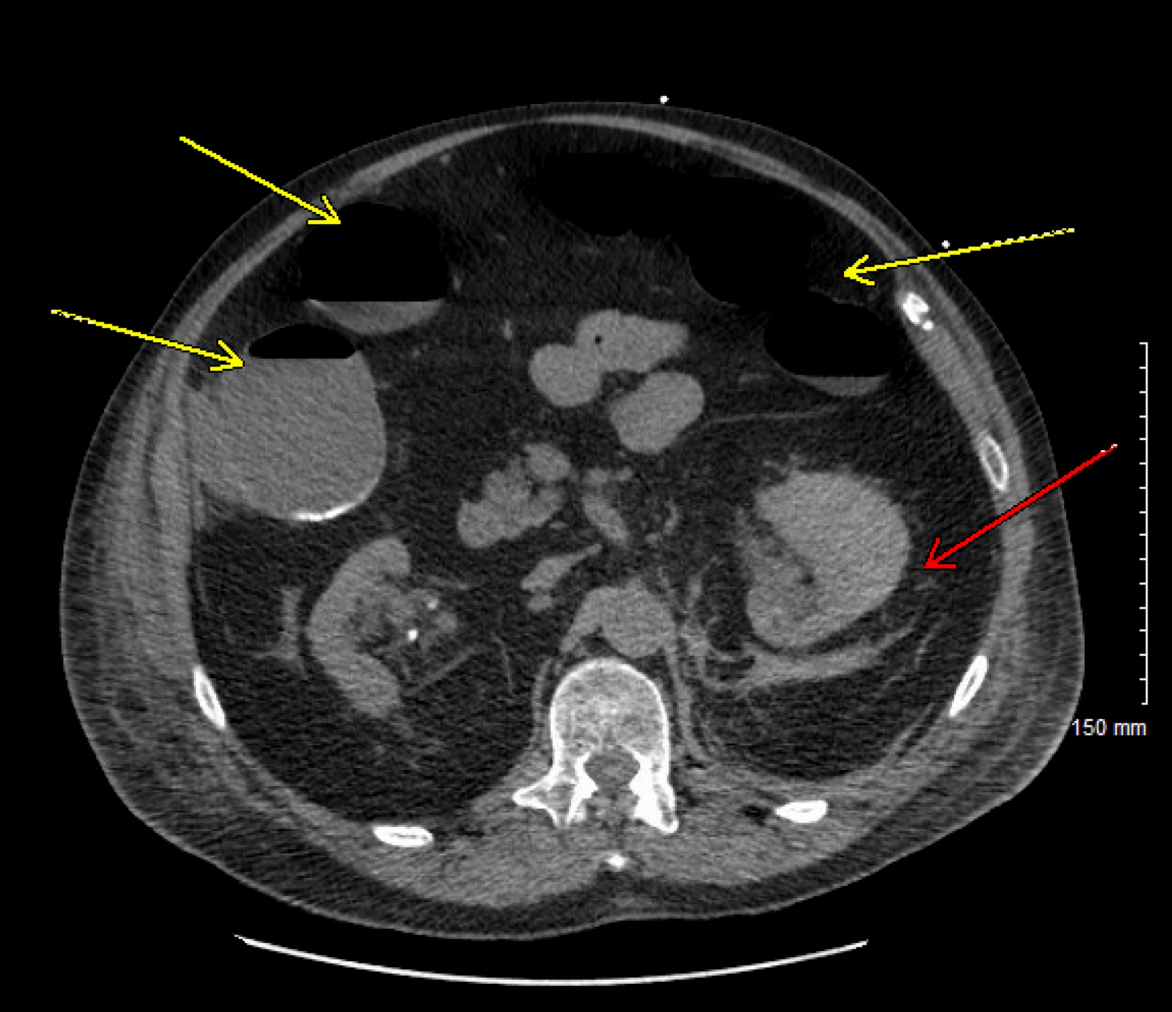
Non-contrast CT Abdomen and Pelvis Demonstrating Persistent Left Perinephric Hematoma (Red Arrow) and Ileus with Dilated Bowel Loops (Yellow Arrows)

On day 6, the patient developed worsening respiratory distress requiring endotracheal intubation and mechanical ventilation, prompting transfer back to the medical ICU. Within minutes of intubation, he became hypotensive with a blood pressure of 79/60 mmHg and a heart rate of 136 beats per minute. Although sedation-related hypotension was considered, the concurrent tachycardia, respiratory rate of 26 breaths per minute, low-grade fever of 100.2°F, and concern for possible infection raised suspicion for evolving sepsis, despite a normal white blood cell count of 6.5 × 10⁹/L (reference range: 4.0-11.0 × 10⁹/L). Broad-spectrum antibiotic (piperacillin-tazobactam) and norepinephrine infusion were initiated. CT abdomen and pelvis (Figure 2) demonstrated persistent perinephric hematoma and dilated bowel loops, with associated air-fluid levels suggestive of paralytic ileus.

Flexible sigmoidoscopy with decompression tube placement was performed on day 7 to relieve presumed intra-abdominal pressure; general surgery and gastroenterology recommended conservative management of the ileus with electrolyte correction, laxatives, and enteral feeds. Intra-abdominal pressures were monitored regularly and remained below the threshold for compartment syndrome.

**Figure 3:**
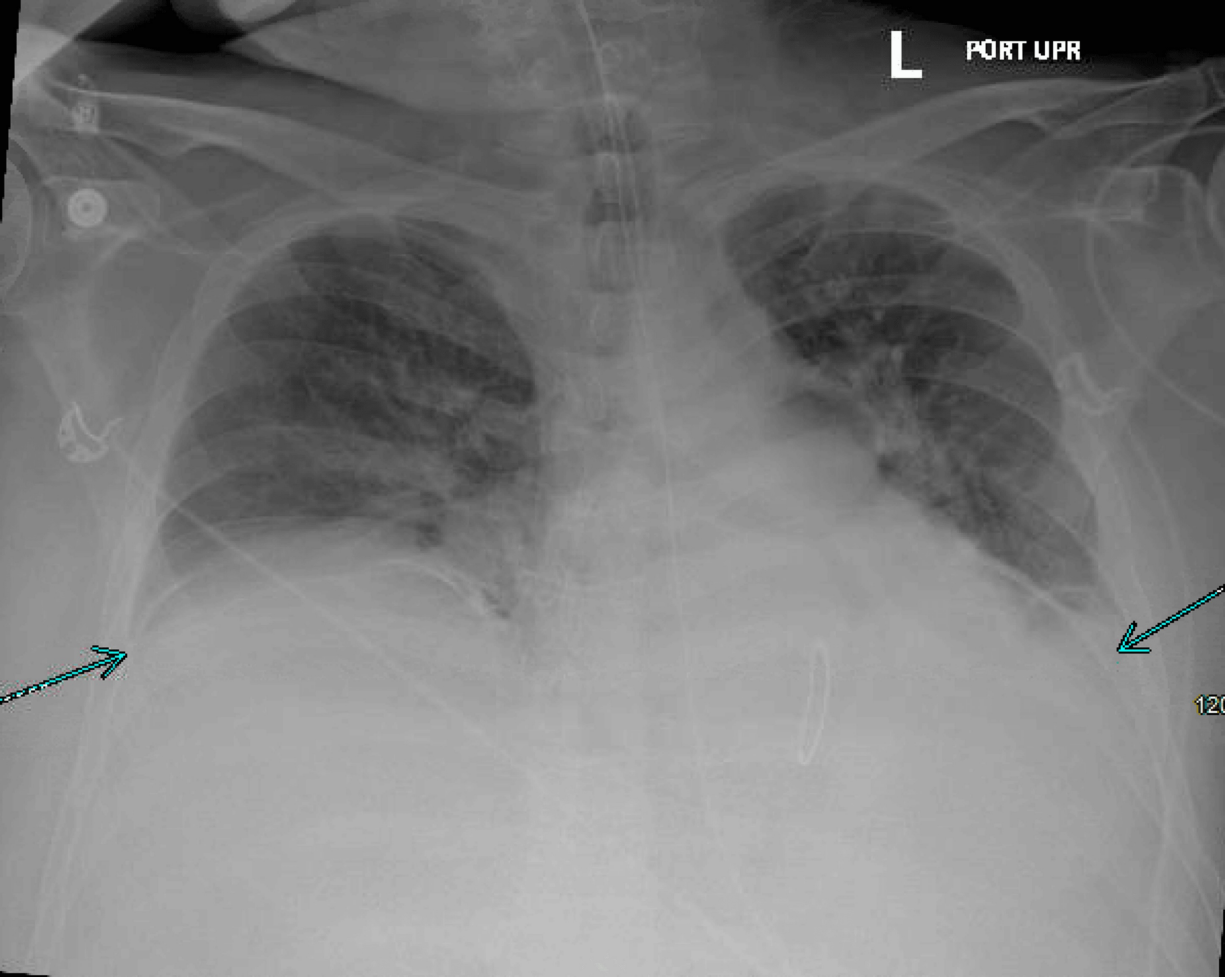
Chest X-ray Showing Small Bilateral Pleural Effusions and Mild Bibasilar Atelectasis, as Indicated by Blue Arrows

Despite these measures, the patient remained ventilator-dependent and required vasopressors for hemodynamic support. Chest X-ray showed small, stable bilateral pleural effusions with mild bibasilar atelectasis without evidence of new infiltrates (Figure 3). Pulmonary embolism was not initially considered due to low clinical suspicion and the absence of supporting features. Transthoracic echocardiogram demonstrated normal left ventricular systolic function with an estimated ejection fraction of 50-55%, normal chamber size, mildly increased wall thickness, and no regional wall motion abnormalities, making a primary cardiac etiology less likely.

On day 9, he developed supraventricular tachycardia with hypotension, requiring synchronized cardioversion and escalation to phenylephrine. Episodes of atrial fibrillation with RVR recurred and were managed with intravenous amiodarone per cardiology input. The renal function continued to decline, and diuretic resistance developed. Hemodialysis was initiated by nephrology. Over the following days, the patient’s clinical status stabilized, with a resolution of septic parameters and discontinuation of vasopressors. However, ventilator weaning trials were unsuccessful, and a tracheostomy and PEG tube were planned. 

**Figure 4:**
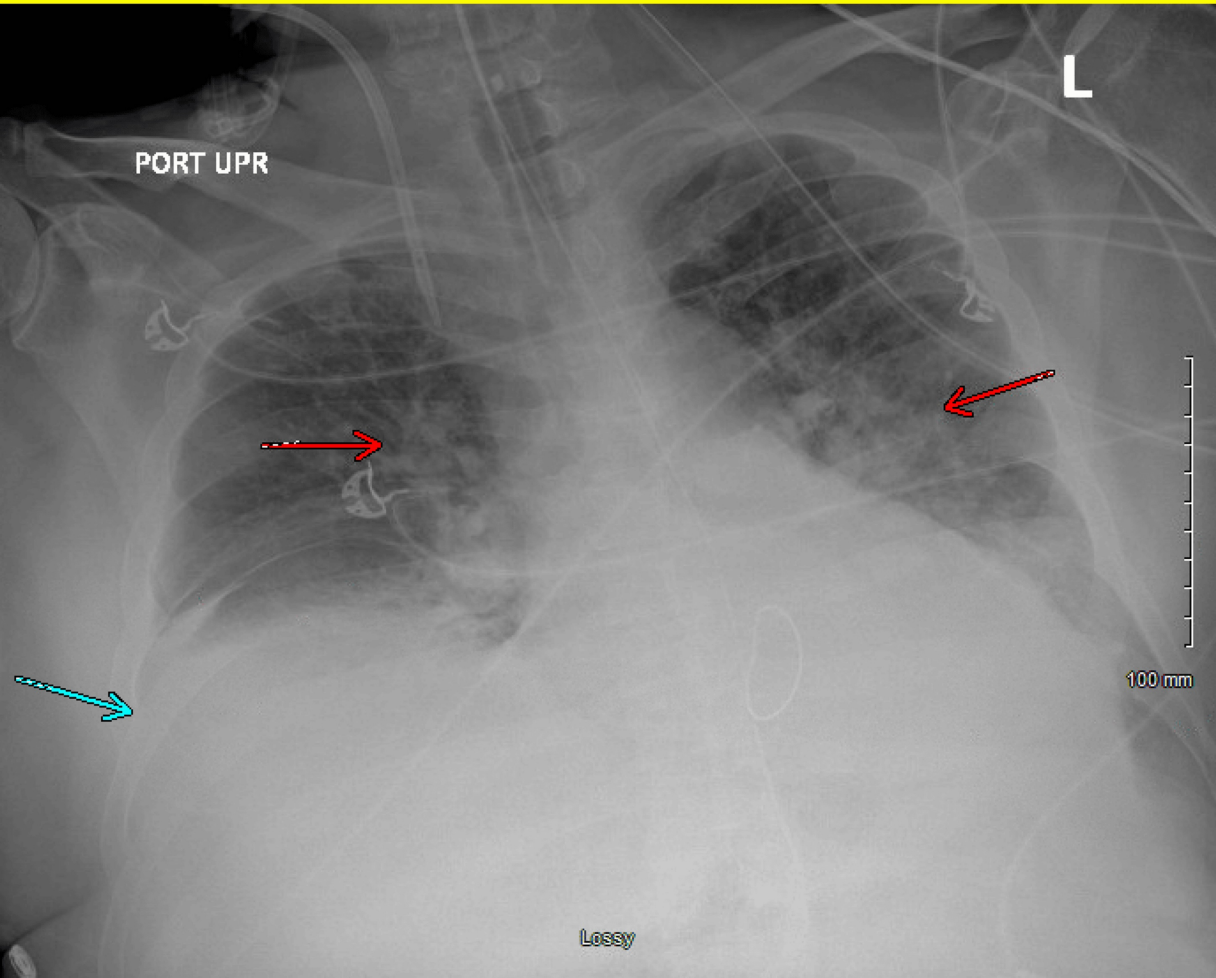
Chest Radiograph Demonstrating Pulmonary Vascular Congestion (Red Arrows) and Pleural Effusion (Blue Arrow)

On day 21 of hospitalization, the patient acutely decompensated. Ventilator peak pressures rose to 50-60 cmH₂O (normal range: 15-30 cmH₂O), and he developed progressive hypoxia. Bronchodilator therapy was administered, and an attempt was made to exchange the endotracheal tube, during which the patient vomited and was subsequently re-intubated. Chest imaging obtained at that time (Figure 4) showed no evidence of pneumothorax but revealed pulmonary vascular congestion and bilateral pleural effusion. This is compared to imaging performed three days earlier (Figure 3), which demonstrated small bilateral effusions and mild bibasilar atelectasis.

Despite maximal ventilatory support and resuscitative efforts, the patient developed pulseless electrical activity and succumbed to refractory hypoxia and cardiac arrest. 

## Discussion

SPH is an uncommon clinical entity, and its diagnosis requires a high index of suspicion, particularly in patients presenting with unexplained flank or abdominal pain and hematuria. In our case, the patient developed classic features of SPH, including acute flank pain and hematuria, without any history of trauma. Imaging with CT is the gold standard for diagnosis, providing critical information on the extent of hemorrhage and potential underlying pathology [1,9]. The etiological spectrum of SPH is broad. Renal tumors, particularly angiomyolipomas and renal cell carcinoma, account for the majority of cases [2]. However, anticoagulation-associated hemorrhage has emerged as an increasingly recognized cause, especially with the widespread adoption of DOACs. A comprehensive review by Gunasekaran et al. emphasized the bleeding risk associated with DOACs, particularly in elderly patients and those with impaired renal function [3]. In our patient, the temporal association between apixaban initiation and presentation strongly supports its role as the precipitating factor. 

Some case reports have described perinephric hematomas associated with apixaban and other factor Xa inhibitors [5-7]. Lee et al. reported a similar case of SPH in a patient on rivaroxaban, managed conservatively with favorable outcomes [5]. In contrast, our patient experienced a significantly more complicated course, likely due to the combination of anticoagulation, baseline cardiovascular disease, and the development of secondary complications. The pathophysiology behind DOAC-induced SPH is not fully understood. Unlike warfarin, which requires routine INR monitoring, DOACs have fixed dosing and fewer monitoring requirements, which can potentially mask the development of coagulopathy. Factor Xa inhibitors impair thrombin generation and clot stabilization, particularly in organs with rich vascular beds like the kidneys [3]. Management of SPH is primarily dictated by the patient's hemodynamic stability and the presence of active bleeding. Conservative management, including discontinuation and/or reversal of anticoagulation, volume resuscitation, and transfusion as needed, is effective in most stable cases [4,9]. Interventional radiology may be consulted for embolization in patients with active arterial bleeding, but success depends on vascular accessibility and the bleeding site. Surgical intervention is reserved for hemodynamically unstable patients or those with expanding hematomas refractory to other measures [9,10]. 

In our patient, both interventional radiology and urology recommended against invasive management due to the risk of nephrectomy and difficulty accessing bleeding vessels. Conservative therapy was pursued, but several cascading organ failures complicated the patient's course. The AKI was likely multifactorial in origin, with contributing factors including hypoperfusion, obstruction from the hematoma, contrast-induced nephropathy, and continued use of an angiotensin receptor blocker. This ultimately necessitated the initiation of renal replacement therapy. Additionally, the patient developed paralytic ileus, likely due to a combination of intra-abdominal hematoma mass effect, inflammation, and opioid-induced hypomotility. Ileus can cause abdominal distention and reduced diaphragmatic excursion, leading to impaired ventilation and hypercapnic respiratory failure, as seen in our case. Ileus and abdominal distention have been associated with elevated intra-abdominal pressures and, in extreme cases, abdominal compartment syndrome, although this was not observed in our patient [9]. 

Recurrent arrhythmias, septic shock, and respiratory failure ultimately culminated in cardiopulmonary arrest. Despite aggressive supportive measures, including hemodialysis, vasopressors, and mechanical ventilation, the patient died from refractory hypoxia and cardiac arrest. This case underscores the high morbidity and mortality associated with anticoagulant-related SPH in medically complex patients [11]. 

## Conclusions

This case highlights the potentially catastrophic consequences of SPH in the setting of DOAC use. In our patient, apixaban-associated SPH led to a cascade of complications, including AKI, paralytic ileus, respiratory failure, septic shock, and ultimately death, despite timely recognition and multidisciplinary management. As the use of DOACs continues to rise, particularly among elderly patients with multiple comorbidities, awareness of this rare but serious complication becomes increasingly critical. This report contributes to the limited but growing body of literature on DOAC-induced SPH. It underscores the importance of maintaining a high index of suspicion in patients presenting with unexplained flank pain, hematuria, or hemodynamic instability. Early diagnosis, individualized treatment strategies, and careful anticoagulant risk assessment are essential to improving outcomes and preventing fatal progression in similar cases. 
